# Lifetime Pesticide Use and Antinuclear Antibodies in Male Farmers From the Agricultural Health Study

**DOI:** 10.3389/fimmu.2019.01476

**Published:** 2019-07-11

**Authors:** Christine G. Parks, Aline de Souza Espindola Santos, Catherine C. Lerro, Curt T. DellaValle, Mary H. Ward, Michael C. Alavanja, Sonja I. Berndt, Laura E. Beane Freeman, Dale P. Sandler, Jonathan N. Hofmann

**Affiliations:** ^1^Epidemiology Branch, National Institute of Environmental Health Sciences, Research Triangle Park, Durham, NC, United States; ^2^Institute in Collective Health Studies, Federal University of Rio de Janeiro, Rio de Janeiro, Brazil; ^3^Occupational and Environmental Epidemiology Branch, Division of Cancer Epidemiology and Genetics, National Cancer Institute, Bethesda, MD, United States; ^4^All of Us Research Program, Office of the Director, National Institutes of Health, Bethesda, MD, United States

**Keywords:** epidemiology–analytic (risk factors), autoantibodies, pesticides, occupational epidemiology, cohort study (or longitudinal study)

## Abstract

Farming and pesticide use have been associated with systemic autoimmune diseases, and while certain organochlorine insecticides and other pesticides are suspected to influence risk, the role of specific pesticides in the development of systemic autoimmunity is not known. We measured serum antinuclear autoantibodies (ANA) by immunofluorescence on Hep-2 cells in 668 male farmers in the study of Biomarkers of Exposure and Effect in Agriculture (BEEA; 2010–2013), an Agricultural Health Study (AHS) subcohort. We examined ANA in relation to lifetime use of 46 pesticides first reported at AHS enrollment (1993–1997) and updated at intervals through BEEA enrollment. Odds ratios (OR) and 95% confidence intervals (CI) were estimated after adjusting for age, state, education, season of blood draw, current pesticide use, and correlated pesticides. Having ANA antibodies (3 or 4+ intensity at a 1:80 dilution, 21% of study participants) was associated with a reported history of seeking medical care due to exposure to pesticides (OR 2.15; 95%CI 1.17, 3.95), use of the fumigant methyl bromide (OR 3.16; 95%CI 1.05, 9.5), and use of petroleum oil/distillates (OR 1.50; 95%CI 1.00, 2.25). Using a higher threshold (3 or 4+ at a 1:160 dilution, 9%) ANA positivity was associated with the carbamate insecticide aldicarb (OR 4.82; 95%CI 1.33, 17.5) and greater combined use of four cyclodiene organochlorine insecticides (top tertile of intensity-weighted lifetime days vs. no use; OR _T3_ 3.20; 95%CI 1.10, 9.27). By contrast, greater use of non-cyclodiene organochlorine insecticides was inversely associated with ANA (1:80 dilution 3 or 4+, OR _T3_ 0.24; 95%CI 0.08, 0.72). Specific autoantibodies (to extractable nuclear antigens and anti-dsDNA), measured on those with ANA detected at the 1:80 dilution 3 or 4+, were seen in 15 individuals (2%), and were associated with use of two or more cyclodiene organochlorine insecticides and several other pesticides (e.g., carbofuran, ethylene dibromide). These findings suggest that specific pesticide exposures may have long-term effects on ANA prevalence and support the hypothesis that certain organochlorine insecticides may increase the risk of developing systemic autoimmunity.

## Introduction

Farming occupation and pesticide use have been associated with elevated risk of systemic autoimmune diseases (1–9), but the role of specific pesticide exposures in the development of systemic autoimmunity is not known. Systemic autoimmunity, as measured by anti-nuclear antibodies (ANA; based on an intensity of 3+ or 4+ at the 1:80 titer), is estimated to occur in 12 to 16% of the US population ages 12 and older, with higher rates in women and older adults (10). Overall ANA positivity, including lower levels of ANA (i.e., at the 1:40 titer), is even more common, seen in almost a third of healthy adults (11). By contrast, higher level ANA (i.e., 1:160 titer and higher) are considered more likely to be clinically relevant; elevated ANA are typically present in patients with systemic lupus erythematosus, an uncommon autoimmune disease, and ANA prevalence is often elevated in family members and patients with other systemic autoimmune diseases (12, 13).

Various pesticides have immunotoxic effects and case series have suggested associations with autoantibodies (14). Only a few epidemiologic studies have examined ANA in relation to specific agricultural pesticides. In a cross-sectional study of Canadian farmers, ANA prevalence was significantly higher in association with use of some, but not all organochlorine insecticides (15). In another similar study from the same region, both positive and inverse associations were seen for ANA with the recent use of different herbicides and fungicides, though estimates were imprecise due to small sample size (16). Other studies have examined serum levels of organochlorine pesticides in relation to ANA, with inconsistent findings (17–19).

In the current study, we examined associations of lifetime pesticide use with ANA in a sample of male farmers from the Biomarkers of Exposure and Effect in Agriculture (BEEA) study in the Agricultural Health Study (AHS) (20), a large prospective cohort that includes farmers in Iowa and North Carolina (21). Cross-sectional studies show that ANA prevalence rises slowly with age over several decades (10, 22, 23). In theory, the loss of immune tolerance enabling the production of antibodies to self-antigens may occur at any time in the past, thereby conferring life-long potential to produce ANA. Therefore, our analyses focused on the association of ANA with lifetime exposures to pesticides, with a specific focus on the organochlorine insecticides.

## Methods

### Study Population and Sample

The design and methodology for enrollment and specimen collection in the AHS and BEEA study have been described (20, 21). BEEA participants were recruited from among the male private pesticide applicators in the AHS who were ≥50 years of age, resided in Iowa or North Carolina, had completed the AHS enrollment questionnaire as well as two follow-up interviews (in 1999–2003 and 2005–2010), and had never been diagnosed with cancer (other than non-melanoma skin cancer). In the current investigation, we assessed ANA and other markers of autoimmunity in 699 participants enrolled in BEEA between June 2010 and September 2013. Because a diagnosis of autoimmune disease might have influenced pesticide use or reporting, we excluded 31 individuals who reported a doctor diagnosis of systemic autoimmune disease at AHS enrollment, for a final analysis sample of 668 participants. The study was approved by Institutional Review Boards at the National Cancer Institute and other participating institutions, and all participants provided written informed consent.

### Questionnaire Data

Lifetime history of 50 specific pesticides was assessed based on participant report at AHS enrollment in 1993–1997. Pesticide use was updated in two follow-up surveys in 1999–2003 and 2005–2010, and at BEEA enrollment. All participants completed these four questionnaires, and 62% (*N* = 410) also completed a “take-home” questionnaire at AHS enrollment that solicited additional detailed information on the use of 28 of the pesticides, including organochlorine insecticides. The enrollment questionnaire also ascertained information about days per year and years of use pesticides overall, along with mixing and application practices, use of protective equipment, and repairing equipment. Data were updated in the follow-up surveys and used to derive intensity-weighted lifetime days of use (24). In the enrollment questionnaire, participants were also asked whether they had ever sought medical care or been hospitalized due to pesticide exposure, and in the take home questionnaire they were asked about high pesticide exposure events (HPEE) and whether they ever had ever been diagnosed with pesticide poisoning (25). Data on HPEE, medical treatment and pesticide poisoning were updated in follow-up surveys and combined as a single variable with three levels: (1) any medical treatment due to pesticides or pesticide poisoning diagnosis, (2) HPEE without medical treatment, and (3) neither. A health history, including diagnosis with rheumatoid arthritis and systemic lupus erythematosus, was collected at enrollment and throughout follow-up. BEEA enrollment questionnaires also collected data on current medication use, which were used to identify use of disease modifying antirheumatic drugs (DMARDs) and non-steroidal anti-inflammatory drugs. Questionnaires are available at https://aghealth.nih.gov/collaboration/questionnaires.html; the primary DMARD medications reported included methotrexate and hydroxychloroquine, as well as leflunomide, sulfasalazine, azathioprine, mycophenolate mofetil, cyclosporine, and biologics, but not steroids.

### Assays

Non-fasting blood specimens were collected in the participant's home and transported and shipped cold via overnight delivery before processing, aliquoting, and storage at −80°C. Antinuclear antibodies (ANA) were measured by the gold standard immunofluorescence assay for serum samples under a standardized protocol in a clinical rheumatology laboratory with prior experience with high-throughput testing in epidemiologic studies (26); in brief, ANA were detected using HEp-2 cell slides (Kallestad, Bio-Rad Laboratories, Hercules, CA), incubated with a 1:80 dilution of sera, then washed and incubated with the burro anti-human polyvalent immunoglobulin FITC conjugate (Kallestad) read using fluorescent microscopy (Leitz Fluorescence Scope, 50/1.0 magnification). Samples with a positive ANA (3 or 4+ at the 1:80 dilution) were subsequently tested for extractable nuclear autoantibodies (ENA), including RNP, SM, SSA (combined 52 and 60 kDa) and SSB (QUANTA Lite, Inova Diagnostics, San Diego, CA; RNP/SM/SSB—ochterlony; SSA—ELISA) and anti-dsDNA antibodies (EIA, Kallenstad). Anti-citrullinated peptide (anti-CCP) antibodies, specific for rheumatoid arthritis (27), were measured by second-generation ELISA; any detectable anti-CCP were seen in 2.3% of the study sample and <1% were positive based on a clinical cut-point of 25 AU/ml, within the expected prevalence in the general population (27). Testing for anti-TPO antibodies was previously described, with antibodies seen in 9% of the study sample (28). Coefficients of variation were <10% for all assays. Frequencies are shown in Supplemental Table 1.

### Analyses

Three cut-points for ANA positivity were considered: first, ANA prevalence was based on a rating of 3+ or 4+ at 1:80 dilution, similar to recently published population-level data on ANA (10). We then lowered the cut-point, by including lower-level ANA, i.e., those with a rating of 2+ at 1:80 dilution, similar to a positive reading at the 1:40 dilution used in prior studies. Finally, we raised the cut-point in analyses limited to those with higher titer ANA based on having 3+ or 4+ intensity rating at the 1:160 dilution. Only five individuals had a positive reading (3+) at the 1:320 level (one with anti-dsDNA antibodies), so these were not considered separately. These groups are labeled as “Any” (≥1:80 2+), “Moderate-higher” (≥1:80 3+) and Higher (≥1:160 3+), to indicate the increasing threshold for positivity. Three sets of models were run comparing the different definitions of ANA positives to those with no detectable ANA, using logistic regression to calculate odds ratios (OR) and 95% confidence intervals (CIs), adjusting for age and other covariates at BEEA enrollment (i.e., BMI, state, education, season of blood draw, and use of agricultural pesticides in the past 12 months). We considered a *p*-value of <0.05 to be statistically significant, and a value of 0.05 to <0.10 as suggestive. Associations were estimated for lifetime use of pesticides (overall, and for use of 46 specific pesticides with at least five ANA positive participants exposed). We also adjusted for correlated pesticides (Spearman's rho ≥0.40 in the sample, Supplemental Table 2) when confounding was observed, i.e., inclusion in the model changed the effect estimate by at least 10%.

We explored intensity-weighted lifetime days for individual organochlorine insecticides (Supplemental Table 3), but numbers were smaller due to a lack of data on those who did not complete the take-home questionnaire, and we saw no clear exposure-response patterns. To increase statistical power, we also grouped the OCs into two groups based on similarities in structure and function. We then tested the hypothesis that ANA was related to use of certain organochlorine insecticides, examining exposure-response relationships across cumulative intensity-weighted lifetime days for two groups of insecticides, summing the intensity-weighted lifetime days for each individual pesticide in the group: cyclodienes (aldrin, dieldrin, chlordane, and heptachlor) and other organochlorines (DDT, toxaphene, and lindane). These two groups were correlated (rho = 0.53 for any use), so they were run in a mutually adjusted model. Finally, we explored the frequency of disease-specific autoantibodies (i.e., ENA and anti-dsDNA autoantibodies) by pesticide use, including combined organochlorine variables (e.g., none, 1, or 2 or more cyclodienes used), and estimated age-adjusted ORs to describe these associations. Sensitivity analyses excluded potential incident cases of autoimmune disease, i.e., based on self-reported rheumatoid arthritis or other autoimmune disease, DMARD use, or the presence of anti-thyroid or anti-citrullinated peptide autoantibodies identified during AHS follow-up or at BEEA enrollment.

## Results

Of 668 samples tested, 282 (42%) had detectable ANA: 143 (21%) had any detectable ANA (i.e., ≥1:80 dilution at 2+ intensity reading), 139 (21%) had ANA detected at ≥1:80 3 or 4+ and 60 (9%) had ANA detectable at 1:160 3 or 4+. Sample characteristics by ANA status are shown in Table 1. Participants with ANA were older: those over age 60 years had two to three times the odds of having moderate to higher-levels of ANA (at ≥1:80 3 or 4+) compared with those ages 50–55 years. After adjusting for age, no associations were noted for smoking status, BMI, state, current farming status and season at the time of blood draw. During follow-up or at BEEA enrollment, 44 participants reported an incident diagnosis of an autoimmune disease, which was associated with overall ANA (OR 3.62; 95%CI 1.87, 6.98) (Supplemental Table 1). Among those with moderate to high level ANA, 15 (2% of the total sample) had disease-specific autoantibodies, i.e., ENA (*n* = 6) or anti-dsDNA (*n* = 11).

**Table 1:** Participant characteristics and age-adjusted associations with ANA.

	**ANA Level**^1^				**Age-adjusted OR (95% CI)**^2^		
**Characteristics**^3^	**Negative *N* = 386**	**1:80 2+ *N* = 143**	**1:80 3/4+ *N* = 79**	**1:160 3/4+ *N* = 60**	**Any ANA (≥1:80 2+) vs. none**	**Moderate-higher ANA (≥1:80 3/4+) vs. none**	**Higher ANA (≥1:160 3/4+) vs. none**
	***N (%)***						
**Age (years)**							
50–55	84 (22)	24 (17)	12 (15)	7 (12)	Referent	Referent	Referent
56–60	102 (26)	34 (24)	14 (18)	5 (8)	1.02 (0.62, 1.67)	0.82 (0.41, 1.66)	0.59 (0.18, 1.92)
61–70	107 (28)	39 (27)	28 (35)	24 (40)	1.66 (1.05, 2.64)	2.15 (1.18, 3.91)	2.69 (1.11, 6.55)
70+	93 (24)	46 (32)	25 (32)	24 (40)	2.00 (1.25, 3.18)	2.33 (1.27, 4.27)	3.10 (1.27, 7.56)
**Smoked**							
Past	120 (31)	53 (37)	30 (38)	36 (60)	1.21 (0.86, 1.70)	1.15 (0.76, 1.77)	1.06 (0.59, 1.92)
Current	14 (4)	8 (6)	4 (5)	3 (5)	1.74 (0.82, 3.67)	1.73 (0.68, 4.45)	NA
**BMI**							
Overweight	166 (43)	74 (52)	38 (48)	24 (42)	1.38 (0.89, 2.16)	1.47 (0.82, 2.64)	1.06 (0.49, 2.32)
Obese	150 (39)	44 (31)	32 (41)	25 (42)	1.17 (0.74, 1.86)	1.58 (0.87, 2.87)	1.32 (0.60, 2.89)
Used agricultural pesticides, past year	288 (75)	100 (70)	54 (68)	39 (65)	0.90 (0.63, 1.30)	0.91 (0.58, 1.44)	0.92 (0.49, 1.71)
Spring/summer^4^	206 (53)	76 (53)	39 (49)	37 (62)	1.03 (0.75, 1.40)	1.07 (0.72, 1.58)	1.44 (0.82, 2.54)
State (NC)	54 (14)	18 (13)	11 (14)	9 (15)	0.93 (0.57, 1.34)	0.88 (0.55, 1.55)	0.88 (0.40, 1.93)

*NC, North Carolina; NA, not applicable due to fewer than five exposed cases*.

1 *ANA Level shows four exclusive categories of ANA positivity based on highest reading observed; Negative, none detected at 1:80 dilution*.

2 *Odds Ratios (ORs) and 95% Confidence Intervals (CI) were calculated by logistic regression models adjusted for age at interview; “Any ANA,” “Moderate-higher ANA,” and “Higher ANA” indicate increasing thresholds for positivity used for modeling*.

3 *All were current at BEEA enrollment, except for state at AHS study enrollment*.

4 *Blood collected in spring or summer, compared to fall or winter*.

Neither lifetime years nor average days per year of any pesticide use at AHS enrollment was associated with ANA positivity (Table 2). However, having sought medical care due to pesticide exposure was associated with having moderate to high level ANA (OR 2.15; 95%CI 1.17, 3.95). In analyses of specific pesticides (Table 3), we saw suggestive associations of high ANA with lifetime use of the organochlorine insecticide heptachlor (OR 1.93; 95%CI 0.96, 3.90) and the organophosphate insecticide diazinon (OR 1.64; 95%CI 0.92, 2.92), and a significant association with the carbamate insecticide aldicarb (OR 4.82; 95%CI 1.33, 17.5). Moderate to high level ANA were associated with the fumigant methyl bromide (OR 3.16; 95%CI 1.05 to 9.50), and suggestively associated with use of petroleum oil/distillates as an herbicide (OR 1.50; 95%CI 1.00, 2.25). By contrast, overall ANA (low, moderate, or high) were inversely associated with the organophosphate insecticides fonofos (OR 0.72; 95%CI 0.50, 1.00) and malathion (OR 0.62; 95%CI 0.42, 0.91), and the herbicide butylate (OR 0.72; 95%CI 0.52, 0.99).

**Table 2:** ANA associations with lifetime pesticide use and high pesticide exposure events.

	**ANA Level**^1^				**Adjusted OR (95% CI)**^2^		
**Overall pesticide use**	**Negative *N* = 386**	**1:80 2+ *N* = 143**	**1:80 3/4+ *N* = 79**	**1:160 3/4+ *N* = 60**	**Any ANA(≥1:80 2+) vs. none**	**Moderate-higher (≥1:80 3/4+) ANA vs. none**	**Higher ANA (≥1:80 3/4+) vs. none**
	***N (%)***						
**MIXED OR APPLIED PESTICIDES – ENROLLMENT**							
**Years**							
≤ 10	65 (18)	22 (16)	12 (16)	9 (15)	Referent	Referent	Referent
11–20	128 (35)	53 (38)	21 (27)	16 (27)	1.07 (0.66, 1.73)	0.88 (0.47, 1.65)	0.92 (0.38, 2.23)
21-30	130 (35)	47 (33)	33 (43)	26 (43)	1.04 (0.64, 1.69)	1.11 (0.61, 2.03)	1.11 (0.48, 2.58)
30+	47 (13)	19 (14)	11 (14)	9 (15)	0.78 (0.41, 1.55)	0.68 (0.31, 1.53)	0.60 (0.20, 1.81)
**Average days/year**							
<10	155 (42)	56 (40)	33 (43)	31 (53)	Referent	Referent	Referent
10–19	125 (34)	49 (35)	11 (14)	13 (15)	0.90 (0.62, 1.31)	0.70 (0.43, 1.14)	0.52 (0.26, 1.05)
20+	89 (24)	36 (25)	18 (23)	15 (25)	1.07 (0.71, 1.60)	0.95 (0.57, 1.57)	0.91 (0.45, 1.81)
**HIGH EXPOSURE EVENTS/MEDICAL CARE/POISONING**^3^							
Yes, but did not seek care	47 (12)	24 (17)	11 (14)	6 (10)	1.37 (0.86, 2.18)	1.22 (0.66, 2.25)	0.94 (0.37, 2.35)
Sought medical care	29 (8)	9 (6)	15 (18)	7 (12)	1.47 (0.85, 2.52)	**2.15 (1.17, 3.95)**	1.41 (0.58, 3.46)

*NA, not applicable due to fewer than five ANA positive individuals who were exposed*.

1 *ANA Level shows four exclusive categories of ANA positivity based on highest reading observed*.

2 Odds Ratios (ORs) and 95% Confidence Intervals (CI) were calculated by multivariable logistic regression models adjusted for age at interview, state, overweight/obese, ever smoked, spring, or summer season, current occupational pesticides use. Bold shows ORs that are statistically significant at p < 0.05.

3 *Ever had a high pesticide exposure event (without seeking medical care for pesticides), or were diagnosed with pesticide poisoning, hospitalized or saw a doctor due to pesticide exposure; referent group is those who did not report a high pesticide exposure event or seeking medical care for pesticide use*.

**Table 3:** Antinuclear antibodies and lifetime use of insecticides.

	**ANA Level**^1^				**Adjusted OR (95% CI)**^2^		
**Pesticide name**	**Negative *N* = 386**	**1:80 2+ *N* = 140**	**1:80 3/4+ *N* = 79**	**1:160 3/4+ *N* = 60**	**Any ANA (≥1:80 2+) vs. none**	**Moderate-higher (≥1:80 3/4+) vs. none**	**Higher (≥1:160 3/4+) vs. none**
	***N (%)***						
**INSECTICIDES**							
**Organochlorines**							
Cyclodienes							
Aldrin^3^	81 (22)	32 (23)	23 (30)	21 (36)	0.96 (0.61, 1.50)	1.21 (0.70, 2.09)	1.08 (0.51, 2.28)
Chlordane	103 (27)	40 (29)	27 (35)	24 (41)	1.16 (0.81, 1.67)	1.35 (0.86, 2.11)	1.44 (0.78, 2.65)
Dieldrin^3^	31 (8)	19 (14)	7 (9)	8 (14)	1.06 (0.59, 1.91)	0.81 (0.39, 1.71)	0.89 (0.34, 2.33)
Heptachlor^3^	70 (19)	37 (27)	18 (24)	20 (35)	1.50 (0.98, 2.29)	1.50 (0.88, 2.54)	1.93 (0.96, 3.90)
Others							
DDT^3^	86 (23)	37 (26)	20 (27)	16 (28)	0.79 (0.51, 1.23)	0.68 (0.39, 1.20)	0.55 (0.25, 1.18)
Lindane	110 (29)	43 (30)	24 (30)	20 (33)	1.04 (0.74, 1.48)	1.07 (0.69, 1.65)	1.12 (0.64, 2.04)
Toxaphene^3^	57 (15)	21 (15)	15 (20)	10 (18)	0.95 (0.58, 1.55)	0.96 (0.53, 1.57)	0.78 (0.48, 1.81)
**Organophosphates**							
Phosphorothioates							
Chlorpyrifos	211 (55)	79 (55)	47 (60)	35 (58)	1.17 (0.85, 1.61)	1.32 (0.87, 1.98)	1.35 (0.76, 2.40)
Coumaphos	39 (10)	16 (11)	9 (11)	8 (13)	1.18 (0.72, 1.95)	1.24 (0.67, 2.31)	1.38 (0.60, 3.16)
Diazinon	112 (29)	50 (35)	22 (28)	26 (43)	1.25 (0.88, 1.76)	1.18 (0.77, 1.83)	1.64 (0.92, 2.92)
Phosphorodithioates							
Dichlorvos	66 (17)	23 (16)	10 (13)	11 (18)	0.85 (0.55, 1.30)	0.80 (0.46, 1.39)	1.02 (0.50, 2.11)
Fonofos	136 (35)	39 (27)	24 (30)	21 (35)	0.72 (0.50, 1.00)	0.80 (0.52, 1.24)	0.93 (0.51, 1.70)
Parathion	53 (14)	20 (14)	10 (13)	6 (11)	0.80 (0.49, 1.28)	0.66 (0.35, 1.22)	0.53 (0.21, 1.33)
Malathion	318 (82)	99 (69)	66 (84)	46 (77)	**0.62 (0.42, 0.91)**	0.87 (0.53, 1.45)	0.67 (0.34, 1.30)
Phorate	158 (41)	57 (40)	30 (38)	21 (35)	0.83 (0.59, 1.16)	0.76 (0.50, 1.17)	0.70 (0.38, 1.27)
Terbufos	212 (55)	70 (49)	42 (53)	29 (48)	0.82 (0.59, 1.14)	0.89 (0.59, 1.34)	0.82 (0.46, 1.46)
**Carbamates**							
Carbofuran	144 (37)	42 (29)	29 (37)	24 (40)	0.79 (0.57, 1.10)	0.95 (0.63, 1.44)	1.02 (0.58, 1.79)
Carbaryl	204 (53)	72 (50)	43 (54)	35 (58)	0.95 (0.69, 1.31)	1.05 (0.70, 1.58)	1.12 (0.63, 1.99)
Aldicarb^3^	20 (5)	7 (5)	6 (8)	7 (12)	1.44 (0.65, 3.18)	2.33 (0.89, 6.11)	**4.82 (1.33, 17.5)**
**Pyrethroids**							
Permethrin	145(38)	55 (39)	34 (43)	16 (27)	1.03 (0.74, 1.44)	1.00 (0.66, 1.53)	0.66 (0.35,1.24)
**HERBICIDES**							
**Phenoxy**							
2,4-D	338 (88)	120 (84)	68 (86)	56 (93)	0.84 (0.52, 1.37)	1.10 (0.57, 2.11)	1.84 (0.61, 5.54)
2,4,5-T	102 (26)	38 (27)	32 (41)	19 (32)	1.10 (0.77, 1.57)	1.33 (0.86, 2.06)	0.97 (0.52, 1.80)
2,4,5-TP	39 (10)	18 (13)	13 (17)	6 (10)	1.27 (0.77, 2.07)	1.27 (0.69, 2.34)	0.81 (0.32, 2.06)
**Triazines**							
Atrazine	331 (86)	125 (88)	71 (90)	54 (90)	1.28 (0.78, 2.07)	1.48 (0.77, 2.84)	1.51 (0.60, 3.84)
Cyanazine	207 (54)	76 (53)	37 (47)	35 (58)	0.91 (0.65, 1.26)	0.88 (0.58, 1.34)	1.20 (0.66, 2.17)
Metribuzin^3^	209 (54)	76 (53)	44 (56)	33 (55)	1.19 (0.82, 1.72)	1.15 (0.72, 1.83)	0.86 (0.44, 1.66)
**Other**							
Chlorimuron ethyl	150 (46)	68 (48)	34 (48)	28 (47)	1.23 (0.89, 1.69)	1.05 (0.70, 1.58)	0.91 (0.51, 1.62)
Metolachlor	221 (57)	78 (54)	41 (52)	38 (63)	0.98 (0.71, 1.35)	1.05 (0.7, 1.58)	1.43 (0.80, 2.58)
Alachlor	247 (64)	88 (62)	56 (71)	41 (68)	1.10 (0.79, 1.53)	1.37 (0.89, 2.10)	1.34 (0.73, 2.43)
EPTC	82 (21)	27 (19)	11 (14)	14 (23)	0.86 (0.58, 1.28)	0.84 (0.50, 1.40)	1.19 (0.61, 2.33)
Butylate	190 (49)	57 (40)	31 (39)	34 (57)	**0.72 (0.52, 0.99)**	0.83 (0.55, 1.25)	1.21 (0.68, 2.15)
Imazethapyr	178 (46)	71 (49)	38 (48)	28 (47)	1.04 (0.74, 1.46)	1.00 (0.65, 1.53)	0.98 (0.53, 1.78)
Dicamba	268 (69)	99 (69)	58 (73)	53 (72)	1.11 (0.75, 1.64)	1.33 (0.79, 2.22)	1.33 (0.64, 2.75)
Paraquat	87 (23)	28 (20)	19 (24)	12 (20)	0.88 (0.59, 1.31)	0.93 (0.57, 1.52)	0.80 (0.39, 1.63)
Glyphosate	353 (92)	129 (90)	73 (92)	54 (90)	0.99 (0.57, 1.73)	1.08 (0.53, 2.21)	0.88 (0.34, 2.26)
Trifluralin	208 (54)	73 (51)	36 (46)	35 (58)	0.84 (0.61, 1.15)	0.81 (0.54, 1.22)	1.05 (0.59, 1.87)
Pendimethalin	193 (50)	70 (49)	44 (56)	29 (48)	1.02 (0.75, 1.41)	1.11 (0.74, 1.66)	0.92 (0.43, 1.62)
Petroleum Oil/Distillates	204 (53)	83 (58)	50 (63)	38 (63)	1.34 (0.97, 1.84)	1.50 (1.00, 2.25)	1.48 (0.83, 2.62)
**FUNGICIDES AND FUMIGANTS**							
Ethylene dibromide^3^	13 (3)	8 (6)	3 (4)	3 (5)	2.06 (0.84, 5.08)	1.93 (0.61, 6.10)	NA
Maneb/Mancozeb^3^	25 (6)	11 (8)	6 (7)	2 (3)	0.85 (0.40, 1.79)	0.50 (0.18, 1.40)	NA
Methyl bromide^3^	25 (6)	9 (6)	8 (10)	7 (12)	1.94 (0.82, 4.60)	**3.16 (1.05, 9.50)**	3.61 (0.79, 16.6)
Aluminum phosphide	23 (6)	16 (11)	4 (5)	3 (5)	1.38 (0.75, 2.53)	0.81 (0.34, 1.96)	NA
Benomyl^3^	26 (7)	10 (7)	4 (5)	2 (3)	0.55 (0.25, 1.21)	0.42 (0.14, 1.22)	NA
Captan	53 (14)	21 (15)	14 (18)	7 (12)	1.06 (0.68, 1.66)	1.08 (0.62, 1.88)	0.79 (0.34, 1.84)
Metalaxyl^3^	74 (19)	25 (18)	16 (20)	12 (20)	0.92 (0.59, 1.44)	0.94 (0.54, 1.64)	0.94 (0.43, 2.05)

*NA, not applicable due to fewer than five ANA positive individuals who were exposed*.

1 *ANA Level shows four exclusive categories of ANA positivity based on highest reading observed*.

2 *Reported ever use at AHS enrollment (1993–1997) based on list of 50 specific pesticides, with updated use throughout follow-up and BEEA enrollment; included in table if at least five ANA positive individuals exposed to individual pesticides*.

3 Odds Ratios (ORs) and 95% Confidence Intervals (CI) were calculated by multivariable logistic regression models adjusted for age at interview, state, overweight/obese, ever smoked, spring or summer season, current occupational pesticides use. Bold shows ORs that are statistically significant at p < 0.05

^4^ *Mutually adjusted for correlated pesticides (see Supplemental Table 2)*.

We examined the relationship of ANA with intensity-weighted lifetime days of use for organochlorines grouped as cyclodienes (heptachlor, chlordane, aldrin, and dieldrin) and non-cyclodienes (DDT, lindane, toxaphene), in a mutually adjusted model (Table 4). Prevalence of high ANA was associated with being in the top tertile of intensity-weighted lifetime days of combined cyclodiene use vs. no use (OR_T3_ 3.20; 95%CI 1.10, 9.27). By contrast, greater use of non-cyclodiene organochlorines was inversely associated with moderate to high ANA (OR_T3_ 0.24; 95%CI 0.08, 0.72), which was similar to the association with greater DDT use (OR 0.35; 95%CI 0.13, 0.99, above the median intensity-weighted lifetime days; Supplemental Table 3).

**Table 4:** ANA associations with intensity-weighted lifetimes days of organochlorine use.

	**ANA Level**^1^				**Adjusted OR (95% CI)**^3^		
**Intensity weighted lifetime days**^2^	**Negative *N = 227***	**1:80 2+ *N = 88***	**1:80 3/4+ *N = 51***	**1:160 3/4+ *N = 40***	**Any ANA (≥1:80 2+) vs. none**	**Moderate-higher (≥1:80 3/4+) vs. none**	**Higher (≥1:160 3/4+) vs. none**
	***N (%)***						
**Cyclodienes ^4^**							
None	151 (66)	57 (65)	32 (63)	20 (50)	1.0 (referent)	1.0 (referent)	1.0 (referent)
Tertile 1	23 (10)	13 (15)	8 (16)	4 (10)	1.29 (0.66, 2.49)	1.40 (0.62, 3.17)	1.27 (0.38, 4.24)
Tertile 2	29 (13)	10 (11)	4 (8)	6 (14)	0.76 (0.38, 1.51)	0.84 (0.36, 1.99)	1.14 (0.38, 3.42)
Tertile 3	24 (11)	8 (9)	7 (14)	10 (25)	1.33 (0.64, 2.75)	**2.58 (1.09, 6.09)**	**3.20 (1.10, 9.27)**
**Other OC ^3^**							
None	147 (65)	55 (63)	35 (69)	26 (65)	1.0 (referent)	1.0 (referent)	1.0 (referent)
Tertile 1	22 (10)	13 (15)	9 (18)	3 (8)	1.23 (0.63, 2.41)	1.14 (0.50, 2.60)	0.62 (0.16, 2.40)
Tertile 2	29 (13)	8 (9)	5 (10)	7 (17)	0.78 (0.41, 1.51)	0.82 (0.37, 1.79)	0.95 (0.35, 2.57)
Tertile 3	29 (13)	12 (14)	2 (4)	4 (10)	0.58 (0.28, 1.23)	**0.24 (0.08, 0.72)**	0.26 (0.07, 1.03)

*NA, not applicable due to fewer than five exposed cases*.

1 *ANA Level shows four exclusive categories of ANA positivity based on highest reading observed*.

2 *Limited to applicators who completed the take-home questionnaire with information on frequency and duration of use*.

3 *Odds Ratios (ORs) and 95% Confidence Intervals (CI) were calculated by multivariable models adjusted for age at interview, state, overweight/obese, ever smoked, spring or summer season, current occupational pesticides use. Bold shows ORs that are statistically significant at p < 0.05*.

4 *ORs for tertiles of cyclodienes (aldrin, chlordane, dieldrin, or heptachlor) and other organochlorines (OCs, DDT, lindane, or toxaphene), mutually adjusted*.

In age adjusted models, having specific ENA or anti-dsDNA autoantibodies was associated with using two or more cyclodiene organochlorine insecticides, and with fonofos, cyanazine, and ethylene dibromide (Table 5). Because these comparisons are based on only a small number of participants with ENA or anti-dsDNA autoantibodies, confidence limits were wide, and we did not adjust these models for other covariates or correlated pesticides. All but one of the participants with disease-specific autoantibodies used carbofuran (*p* = 0.002). We also saw suggestive associations of disease-specific autoantibodies with aldicarb and methyl bromide, and an inverse association with malathion. Sensitivity analyses showed no substantial impact of excluding participants with a potential autoimmune disease during follow-up or at BEEA enrollment, i.e., who reported an incident diagnosis of an autoimmune disease since enrollment, or used disease-specific antirheumatic drugs, or were positive for thyroid or anti-CCP autoantibodies (not shown).

**Table 5:** ENA and anti-dsDNA autoantibodies by lifetime pesticide use^1^.

	**ANA Negative (*****N*** **=** **386)**		**ENA/anti-dsDNA (*****N*** **=** **15)**		**Age-adjusted odds ratio (95% CI)**^2^			
	***N***	**%**	***N***	**%**				***p*-value**
**ORGANOCHLORINE INSECTICIDES**								
**Cyclodienes**								
Aldrin	81	22	9	60	3.39	(0.98, 11.7)		0.054
Chlordane	103	28	8	53	1.72	(0.55, 5.36)		0.350
Dieldrin	31	8	5	33	3.29	(0.88, 12.3)		0.076
Heptachlor	70	19	7	47	2.86	(0.80, 10.2)		0.106
Only one	78	21	2	13	1.39	(0.21, 9.08)		0.729
2 or more	74	20	10	67	**6.48**	**(1.45, 29.0)**		0.015
**Others**								
DDT	87	23	7	47	1.06	(0.29, 3.84)		0.934
Lindane	110	29	8	53	2.27	(0.77, 6.71)		0.140
Toxaphene	57	15	3	21	0.76	(0.18, 3.19)		0.708
One	103	27	2	13	0.45	(0.08, 2.41)		0.352
2 or more	65	17	7	47	1.74	(0.47, 6.43)		0.403
**OTHER INSECTICIDES**								
Chlorpyrifos	211	55	5	33	0.45	(0.14, 1.39)		0.166
Coumaphos	39	10	1	7	0.57	(0.07, 4.74)		0.607
Diazinon	112	29	6	40	1.20	(0.39, 3.71)		0.746
DDVP	66	17	5	33	2.12	(0.64, 7.01)		0.218
Fonofos	136	35	9	60	**3.70**	(**1.01, 13.5**)		0.048
Parathion	53	14	3	20	0.88	(0.22, 3.55)		0.863
Malathion	318	82	9	60	0.31	(0.10, 0.95)		0.041
Phorate	158	41	9	60	2.63	(0.70, 9.88)		0.152
Terbufos	212	55	10	67	3.32	(0.87, 12.6)		0.078
Carbofuran	144	37	14	93	**30.0**	(**3.59, 251**)		0.002
Carbaryl	204	53	10	67	1.41	(0.44, 4.55)		0.562
Aldicarb	20	5	3	20	5.28	(0.95, 29.5)		0.058
Permethrin	145	38	3	20	0.41	(0.11, 1.56)		0.190
**HERBICIDES**								
2, 4-D	338	88	14	93	1.77	(0.21, 15.2)		0.604
2, 4, 5-T	102	26	7	47	1.47	(0.47, 4.63)		0.512
Atrazine	331	86	14	93	2.44	(0.28, 21.4)		0.421
Cyanazine	207	54	12	80	**5.34**	(**1.12, 25.4**)		0.035
Metribuzin	209	54	9	60	1.21	(0.37, 3.89)		0.754
Chlorimuron ethyl	150	39	7	47	1.23	(0.41, 3.67)		0.713
Metolachlor	221	57	9	60	1.20	(0.39, 3.66)		0.745
Alachlor	247	64	10	67	1.47	(0.44, 4.96)		0.532
EPTC	82	21	5	33	2.42	(0.73, 8.06)		0.149
Trifluralin	208	54	9	60	1.09	(0.35, 3.42)		0.882
Butylate	190	49	9	60	1.77	(0.56, 5.60)		0.330
Imazethapyr	178	46	9	60	3.02	(0.79, 11.6)		0.107
Dicamba	268	69	11	73	1.78	(0.37, 8.55)		0.474
Paraquat	87	23	4	27	0.95	(0.27, 3.31)		0.941
Glyphosate	353	92	12	80	0.41	(0.10, 1.65)		0.209
Pendimethalin	193	50	9	60	2.02	(0.66, 6.24)		0.220
Petroleum_oil	204	53	10	67	1.82	(0.56, 5.90)		0.321
**FUNGICIDES, FUMIGANTS**								
Benomyl	26	7	3	20	1.92	(0.42, 8.73)		0.397
Captan	53	14	3	20	1.61	(0.42, 6.18)		0.489
Metalaxyl	74	19	5	33	1.74	(0.52, 5.88)		0.372
Methyl bromide	25	6	3	20	16.6	(0.92, 299)		0.057
Aluminum Phosphide	23	6	2	13	2.20	(0.44, 11.0)		0.337
Ethylene Dibromide	13	3	3	20	**5.07**	(**1.10**, **23.4**)		0.038

*ENA (Extractable Nuclear Antigens) measured on those ANA read as 3 or 4+ at 1:80 dilution; ANA Negative = none detected at 1:80 dilution*.

1 *Reported ever use at AHS enrollment (1993–1997) based on list of 50 specific pesticides, with updated use throughout follow-up and BEEA enrollment*.

2 *Odds Ratios (ORs) and 95% Confidence Intervals (CI) were calculated by multivariable logistic regression models adjusted for age at interview*.

## Discussion

Our findings provide evidence that moderate to higher level ANA are associated with past use of specific pesticides and a history of seeking medical care for pesticide exposures in male farmers. Further, our results support the hypothesis that certain types of organochlorine insecticides may increase risk of developing autoimmunity. Two other pesticides, the carbamate insecticide aldicarb, and fumigant methyl bromide, were associated with increased odds of having moderate or higher-level ANA. By contrast, two of more commonly used organophosphate insecticides (in this sample), malathion and fonofos, and the herbicide butylate, were related to lower overall ANA. Together, these results suggest complex and lasting effects of some pesticides on autoimmunity.

Systemic autoimmune diseases are more common in reproductive age women, so past studies have been interested in the potential endocrine-disrupting effects of organochlorine pesticides on autoimmunity. Experimental and epidemiologic studies suggest a nuanced relationship. For example, the insecticide chlordecone (a cyclodiene) accelerated in a dose-dependent fashion the development of disease-specific anti-dsDNA autoantibodies in ovariectomized female mice in the NZB X NZW(F1) model of lupus (29). However, these effects did not appear to be mediated by estrogen-related mechanisms and did not extend to DDT. A recent study of a U.S. population sample (*N* = 4,340; National Health and Examination Survey, 1999–2004) reported no significant associations of measured organochlorines with moderate or higher ANA (3+ or 4+ at the 1:80 titer) after adjusting for multiple comparisons (17); in men, however, ANA was negatively associated with the DDT metabolite, DDE, and oxychlordane (a chlordane metabolite) at the *p* < 0.05 level. By contrast, in a small sample of African-American male farmers, higher DDE levels were suggestively (but non-significantly) associated with ANA (1:40 titer, equivalent to “overall” in the current study) (19). This later sample was selected from the population of rural eastern North Carolina as an add-on study to the AHS (non-overlapping with the current study sample); participants had an average of 2.2 (SD 5.5) years of DDT use and 12.3 (SD 13.4) years of overall pesticide use. In the current study sample, prevalence of high or moderate ANA was associated with greater intensity-weighted lifetime days using any of the four cyclodiene organochlorine insecticides (i.e., aldrin, dieldrin, chlordane, and heptachlor), and inversely associated with greater use of the non-cyclodiene organochlorine insecticides (especially DDT). Notably, DDT and the cyclodiene insecticides are no longer in use in the U.S., but our results suggest persistent effects on immunity decades later.

Our analyzing cyclodienes separately from the other organochlorines had both empirical and theoretical rationale. In analyses of ever-use of the individual organochlorine insecticides, we saw suggestive positive associations with heptachlor and chlordane, but not with DDT. This was consistent with results by Rosenberg et al. who reported that ANA prevalence was associated with lifetime organochlorine use, *excluding* DDT and methoxychlor in a sample of 322 rural adults (15). Organochlorine insecticides share a chlorinated hydrocarbon structure and physical properties such as lipid solubility and environmental persistence. Their toxic effects on insects are due to hyperexcitation of nerve cells. The DDT-type chemicals work by perturbing sodium gates, while the cyclodiene insecticides impact the γ-aminobutyric acid (GABA) receptor, impairing chlorine influx (30). While GABA is best known as a neurotransmitter, emerging evidence suggests an important role in immune function and innate immunity (31, 32). The closely related cyclodienes, chlordane, and heptachlor, have been associated with ANA in case series (33). Similar studies of aldrin and its breakdown product, dieldrin, were not identified in the autoimmunity literature, but there is some evidence of immunosuppressive effects of aldrin/dieldrin across different studies (34–36). In our data, having used two or more of these pesticides was also associated with having any of the disease-specific autoantibodies (e.g., anti-dsDNA autoantibodies). Together, these findings suggest these pesticides may increase the risk of developing clinically relevant autoimmunity. Lindane, a cyclohexane, also impacts GABA receptor function, but we saw limited evidence of an association with ANA. Besides their neurotoxicity, organochlorine pesticides can have other effects, for example endocrine disruption through estrogen or pregnane X receptors (37, 38), so targeted research is needed to identify which pathways may impact autoimmunity.

The carbamate insecticide aldicarb was the only other insecticide associated with high ANA; although it has a high acute toxicity, its immune effects are not well-known (39–41). The other carbamate insecticides, carbaryl and carbofuran, were not associated with elevated ANA. However, all but one of the participants with disease-specific autoantibodies had used carbofuran. High ANA was suggestively associated with the organophosphate insecticide diazinon, and we noted increased ORs for the two other phosphorothioates, chlorpyrifos, and coumaphos, though neither was statistically significant. These chemicals have documented immunotoxic effects (42–45). Conversely, the phosphoro*di*thioate insecticides fonofos and malathion were inversely associated with overall ANA, and the other phosphoro*di*thioate insecticides also showed inverse, though not statistically significant, associations with overall ANA. There is some evidence of immunosuppression by malathion (46), while other studies (including in a MRL-lpr lupus mouse model) suggest that malathion may stimulate and accelerate the onset and intensity of the autoimmune response (47, 48). Immune effects of fonofos are not described in the literature, and our findings seem somewhat paradoxical; while inversely associated with overall ANA, fonofos was positively associated with disease-specific autoantibodies in those with moderate to high ANA compared with no ANA detected. The primary mechanism of action for organophosphate and carbamate insecticides is blocking acetylcholinesterase enzyme. Given cholinergic receptors on human lymphocytes, pesticides may influence the immune response through acetylchlolinergic pathways, though the direction of effects may depend on the activated pathway (49, 50). The phosphoro*di*thioates require oxidative activation *in vivo*. However, it is unclear how this would differentially influence immune-related mechanisms of action compared with the other organophosphates and carbamates.

We also saw an association of high or moderate level ANA with the fumigant methyl bromide. Ethylene dibromide was also associated with disease-specific autoantibodies. Immune-related mechanisms of these highly toxic chemicals are not well-described in the literature, although one study suggested a similar threshold for immune modulation and the level at which acute toxicity occurred (51, 52). Along with aldicarb and carbofuran, methyl bromide and ethylene dibromide are often used against nematodes. Many participants with elevated ANA and disease-specific autoantibodies used more than one of these chemicals, but we did not have sufficient sample size to examine them independently. Use of petroleum oil or distillates was also associated with high or moderate ANA. Petroleum oil is a plausible candidate for inducing systemic autoimmunity, given the model of pristane-induced lupus (53). Pristane is a byproduct of petroleum distillation and induces lupus-like autoantibodies and clinical disease in mice. Petroleum distillates in the past may also have included other contaminants such as benzene or xylene. Overall ANA prevalence was inversely associated with butylate, a thiocarbamate herbicide, linked to lymphohematopoetic cancers and NHL in the AHS (54), but immune effects are not described in the literature. Interestingly, past use of butylate and recent use of malathion were associated with shorter leukocyte telomere length, a potential marker of immune aging, in a different subsample of AHS participants (55).

Our results also suggest that seeking medical care due to pesticide exposure (i.e., seeing a doctor or being hospitalized due to pesticide exposure, or being diagnosed with pesticide poisoning) was associated with having moderate or high ANA. The reasons for seeking medical care for pesticide exposure included high pesticide exposure events (HPEE) and presumably pesticide-related symptoms, however we did not analyze ANA in relation to symptoms data. We also saw an elevated OR for overall ANA with HPEE in those who experienced an event but did not seek medical care (OR 1.37). Prior AHS research suggests those reporting high pesticide exposure events used pesticides for more days per year and had certain practices that might increase personal exposures, such as storing pesticides in the home or spraying pesticides with open cab windows, and that many do not seek medical care for HPEE and exposure-related symptoms (56). In our study, among ANA positive individuals, only 21 (3% of the total) reported both HPEE and seeking medical care, while 39 (6%) sought medical care without reporting HPEE, and 88 (13%) reported only HPEE (not shown in tables). Thus, the actual exposures underlying our findings remain undetermined. The AHS collected data on specific pesticides involved in some high pesticide exposure events, but we had insufficient sample size to take advantage of these data.

Diverse and interrelated pathways lead to dysregulation of the immune response and development of autoimmunity. In healthy individuals, ANA may reflect increased exposure to self-antigens and dysregulation of various pathways, some of which are shared and others distinctive from patterns seen in autoimmune disease patients (57, 58). Pesticides may influence both the generation of autoimmunity and the strength of the immune response. Our study used the gold standard assay, which is sensitive to lower levels of ANA at the 1:40 dilution level while providing increased specificity at higher titers (e.g., 1:160). ANA are thought to arise as an immune response to nuclear antigens released from apoptotic cells. The interpretation of ANA detection at different thresholds for positivity varies by context: although detection at the 1:80 dilution of 3 or 4+ intensity has recently been specified among the constellation of clinical criteria for systemic lupus erythematosus, higher levels may be seen in lupus and sometimes associated with other clinical autoimmune diseases (59). Interestingly, we only saw positive associations for moderate or higher-level ANA, while inverse associations were seen only with having any detectable ANA (including lower levels, at least 2+ intensity at the 1:80 dilution).

A positive response to the HEp-2 ANA assay does not imply an association with clinical disease. The pattern of nuclear antibody staining is emerging as a key means to differentiate the origins and relevance of ANA in clinical and healthy populations, especially the dense fine speckled pattern and the associated anti-DFS70 antibody (60–62). Our analyses did not consider the fluorescence pattern, as the vast majority of ANA were identified as fine speckled, which at the time of the study was not differentiated from dense fine speckled and speckled. Antibodies to DFS70 may be a promising marker, as they appear to be more common in the general population and may even help to rule-out specific autoimmune diseases (60); had use of this marker been more common and validated at the time of the original testing, it would have been considered. We focused on specific disease-associated autoantibodies commonly examined in clinical assessments for SLE, but our results support further investigation of other types of antibodies as well, such as anti-histone and chromatin, which have been linked with certain drug- and xenobiotic exposures and may help to identify the underlying role of specific exposures (63–66).

Half of the ANA-positive individuals in our study sample were positive only at the lowest-level (2+ at a 1:80 dilution); this threshold was used to enable comparisons with studies using a lower dilution (i.e., 1:40). The implications of having lower-level ANA are not well-understood. Most research on autoimmunity in the absence of autoimmune diseases has been based on cross-sectional and clinical samples; however, a few longitudinal studies showing that individuals with low level ANA are more likely to revert to seronegative status over time compared to those at lower titers (22, 62, 67). Notably, in a sample of rural residents and farmers, Semchuk observed that ANA prevalence at higher titers (3+ or 4+ at 1:80 and 1:160) was similar in the summer and winter, with similar rates of seroconversion and reversion across the seasons (16). On the other hand, lower level ANA (at the at 1:40 dilution, but not higher) were elevated in summer, supporting the idea that changing exposures by season may account for transient expression of ANA in some individuals. Although we adjusted for season in our statistical models, we saw no strong or consistent differences in ANA frequency for participants sampled at different times of year. Smoking history and BMI at the time of sampling were non-significantly associated with ANA and were included in our multivariable models along with other covariates as they generally increased the precision of observed associations. The causal relationship of specific pesticides with BMI is unknown; however, adjusting for BMI did not appear to explain any of the observed pesticide associations. Odds ratios approximate the relative risk for rare outcomes (<10%), for example with high ANA and ENA, but overestimate the relative risk for more common outcomes (i.e., overall ANA or high/moderate ANA). Our estimated ORs, therefore, may most accurately reflect the relative risks for high ANA. On the other hand, small numbers may inflate the OR and confidence limits, for example, in the analyses of uncommon pesticides and disease-specific autoantibodies.

We excluded prevalent cases of autoimmune diseases (e.g., rheumatoid arthritis and lupus) to reduce potential recall or reporting bias, or changes in exposures due to health conditions; in follow-up, some participants reported incident autoimmune diseases, which were associated with ANA as expected (Supplemental Table 1). Overall ANA (i.e., including low level ANA) was also associated with anti-TPO autoantibodies. Autoimmune thyroid diseases occur more often in those with systemic autoimmune diseases (68), and anti-TPO autoantibodies have been previously associated with ANA in healthy controls (57). Prevalence of anti-CCP autoantibodies, specific for rheumatoid arthritis, was not associated with ANA in our sample, and levels were similar to the general population (69–71), despite evidence that rheumatoid arthritis may be associated with agricultural work and pesticide use in the AHS and other studies (1–9). Sensitivity analyses showed no substantial changes after excluding possible new cases of autoimmune diseases and those specific autoantibodies (i.e., anti-CCP, anti-TPO, and disease specific ENA and anti-dsDNA autoantibodies) (not shown).

The BEEA study provides an opportunity to investigate the long-term health effects of pesticides and other agricultural exposures on autoimmunity. The study sample is representative of men who were actively farming at AHS enrollment in the mid-1990s, most of whom did not have a diagnosis of RA or other autoimmune diseases. Women in the AHS, mostly spouses of the licensed applicators, were not included in the BEEA sample. Spouses reported less pesticide use than the applicators, but potential associations of pesticides with ANA in spouses may be relevant given the higher prevalence of ANA in women generally. Farmers are a unique population, with diverse exposures to agricultural pesticides and other physical or chemical factors, so results may not be generalizable to the general population. Further research is warranted to investigate ANA in relation to other agricultural factors, for example to solvents and fertilizers, recently associated with rheumatoid arthritis in the AHS (72). Our study addresses several limitations of prior studies on pesticides and ANA, with data on lifetime exposure to over 40 specific agricultural pesticides. However, despite having a larger sample than most studies, we had low statistical power to examine associations with uncommon pesticides and disease-specific autoantibodies, and for testing exposure-response gradients. Non-differential exposure misclassification is a possibility due to errors in recall of past exposures. Evaluation of self-reported data in the AHS has established the reliability of recall for specific pesticides; further, an intensity-weighting algorithm developed for the study (integrating across multiple determinants of exposure intensity) has shown good correlation with measurement data in an exposure sub-study, and may reduce the potential for non-differential misclassification to bias results toward the null in analyses of dose-response (24, 73, 74).

Our analyses are based on prospectively collected exposure data, but temporal ambiguity relative to ANA incidence is inherent to our study design focused on lifetime exposures. We cannot know whether exposures occurred prior to or after the development of ANA, or whether ANA appeared in the past and became non-detectable at the time they were measured. While the natural history of ANA in the general population is not well-understood, ANA prevalence typically increases with age, suggesting increasing incidence of ANA over time. Presumably, pesticides may influence two processes that contribute to the observed ANA at a single time point: (1) the breach of immune tolerance and initial production of autoantibodies, and (2) external factors impacting the production of measurable levels at the time of sampling. Further research is needed to investigate the associations of ANA with contemporary use of specific pesticides and the time since last use. Autoantibodies can precede the development of autoimmune diseases for a decade or longer (75), so other factors are likely to contribute to the development of pathology and disease.

Altogether our findings provide evidence that past pesticide use may influence the development of autoimmunity in middle age and older farmers, consistent with prior findings of elevated ANA with some organochlorine insecticides. The presence of disease-specific autoantibodies was also related to certain organochlorines, supporting the need to further explore these persistent pesticides as potential causal agents in development of autoimmune diseases. Future research should focus on the role of recent exposure and the evaluation of less common pesticides in larger studies or in targeted populations with greater use of these chemicals.

## Data Availability

Due to ethical restrictions imposed in the interest of protecting participant confidentiality, the datasets for this study are available upon request to interested, qualified researchers. Requests to access the datasets should be directed to JH (hofmannjn@mail.nih.gov).

## Ethics Statement

The study was approved by Institutional Review Boards at the National Cancer Institute and other participating institutions, and all participants provided written informed consent.

## Author Contributions

CP conceived of the research question, obtained funding for assays, performed analyses, evaluated results, and wrote the manuscript. AS assisted with planning analyses, evaluated results, and contributed to the manuscript. SB, MW, and CL assisted with planning analyses, evaluating results, and editing the manuscript. JH, DS, MA, CD, and LB obtained funding and generated data for the AHS and BEEA sub-cohort, assisted with planning analyses, evaluated results, and edited the manuscript.

### Conflict of Interest Statement

The authors declare that the research was conducted in the absence of any commercial or financial relationships that could be construed as a potential conflict of interest.
