# Supplementary material for: Lifetime Pesticide Use and Antinuclear Antibodies in Male Farmers From the Agricultural Health Study
Source: Front Immunol. 2019 Jul 11;10:1476. doi: 10.3389/fimmu.2019.01476 (PMC6637299; doi:10.3389/fimmu.2019.01476)
Supplement: Supplementary file 1 [file Table_1.DOCX]

Supplemental Table 1. Autoimmune-related conditions, medications and antibodies: age-adjusted associations with ANA antibodies

|  | ***ANA N (%)*** | | | |  | ***Age-adjusted Odds Ratio (95% CI)^2^*** | | |
| --- | --- | --- | --- | --- | --- | --- | --- | --- |
| ***Characteristics^1^*** | Negative  N=386 | Low  N=143 | Moderate  N=79 | High  N=60 |  | Any ANA  vs. none | High or moderate ANA vs. none | High ANA  vs. none |
| Autoimmune disease | 13 (4) | 9 (6) | 5 (13) | 17 (28) |  | 3.62 (1.87, 6.98) | 6.95 (3.37, 14.3) | 9.62 (4.39, 21.10) |
| Current medications |  |  |  |  |  |  |  |  |
| DMARDs | 3 (1) | 6 (4) | 1 (1) | 3 (5) |  | 4.63 (1.25, 17.1) | 3.71 (0.81, 17.0) | 6.65 (1.28, 34.4) |
| NSAIDs | 22 (6) | 14 (10) | 8 (10) | 6 (10) |  | 1.75 (0.97, 3.14) | 1.75 (0.86, 3.56) | 1.72 (0.66, 4.47) |
| Corticosteroids | 13 (3) | 4 (3) | 1 (1) | 1 (2) |  | 0.59 (0.22, 1.57) | NA | NA |
| Autoantibodies |  |  |  |  |  |  |  |  |
| Anti-CCP2 | 9 (2) | 8 (6) | 0 (0) | 1 (2) |  | 1.36 (0.53, 3.61) | NA | NA |
| Anti-TPO – moderate  Anti-TPO – strong | 13 (3)  18 (5) | 4 (3)  13 (9) | 2 (3)  5 (6) | 4 (7)  4 (7) |  | 1.11 (0.47, 2.58)  1.90 (0.99, 3.65) | 1.35 (0.50, 3.68)  1.61 (0.69, 3.72) | NA  NA |
| ENA and anti-dsDNA**^3^** | --- | --- | 3 (8) | 12 (20) |  | --- | --- | --- |

Any = high, mod or low ANA; high = 3+ or 4+ at 1:160 dilution; Moderate = 3+ or 4+ at 1:80 only; Low =2+ at 1:80

^1^Autoimmune disease self-reported during AHS questionnaires or at BEEA diagnosis; Prescription medications only, DMARDs = disease modifying antirheumatic drugs; NSAIDs = non-steroidal anti-inflammatory drugs; CCP-2 = anti-citrullinated peptide, TPO=thyroperoxidase, ENA=antibodies to extractable nuclear antigens and anti-dsDNA= antibodies to double stranded DNA

^2^Odds Ratios (ORs) and 95% Confidence Intervals (CI) were calculated by multivariable logistic regression models adjusted for age at interview, state, overweight/obese, ever smoked, spring or summer season, current occupational pesticides use. NA=not applicable due to fewer than 5 exposed cases

^3^Tested only on moderate or high ANA.

Supplemental Table 2. Correlated pesticides considered as potential confounders

| Main effect | Correlated pesticides | |
| --- | --- | --- |
| **Insecticides** |  |  |
| Aldrin | Dieldrin | Heptachlor |
| Dieldrin | Heptachlor | Chlordane |
| Heptachlor | Dieldrin | Aldrin |
| Chlordane | Dieldrin | Toxaphene |
| Aldicarb | Maneb | Methylbromide |
| **Herbicides** |  |  |
| Trifluralin | Butylate | Metribuzin |
| Metribuzin | Butylate |  |
| **Fungicides** |  |  |
| Maneb | Ethylene dibromide | Benomyl |
| **Fumigants** |  |  |
| Benomyl | Ethylene dibromide |  |

All pairs with Spearman rho>0.40

Adjustment when >10% change to main effect estimate

Supplemental Table 3. Lifetime use of individual organochlorine insecticides, exposure-response for individual organochlorines

|  |  | **ANA Negative** | | **ANA any (low to high)** | | | | | **ANA Moderate or High** | | | | | **ANA High** | | | | |
| --- | --- | --- | --- | --- | --- | --- | --- | --- | --- | --- | --- | --- | --- | --- | --- | --- | --- | --- |
|  |  | (N=386) | | (N=178) | | | | | (N=90) | | | | | (N=40) | | | | |
| Exposure^1^ |  | N | % | N | % | OR^2^ | 95% CI | | N | % | OR^2^ | 95% CI | | N | % | OR^2^ | 95% CI | |
| Aldrin | None | 184 | 81 | 136 | 77 | Ref |  |  | 64 | 73 | Ref |  |  | 24 | 65 | Ref |  |  |
|  | M1 | 12 | 5 | 15 | 9 | 1.26 | 0.55 | 2.90 | 8 | 9 | 1.58 | 0.58 | 4.26 | 4 | 11 | NA |  |  |
|  | M2 | 14 | 6 | 14 | 8 | 1.05 | 0.46 | 2.38 | 9 | 10 | 1.54 | 0.60 | 4.00 | 6 | 16 | 2.66 | 0.85 | 8.30 |
| Chlordane | Never | 187 | 83 | 138 | 79 | Ref |  |  | 68 | 77 | Ref |  |  | 26 | 70 | Ref |  |  |
|  | M1 | 15 | 7 | 23 | 13 | 1.79 | 0.88 | 3.65 | 11 | 13 | 1.76 | 0.75 | 4.13 | 5 | 14 | 1.92 | 0.62 | 5.89 |
|  | M2 | 24 | 11 | 14 | 8 | 0.69 | 0.33 | 1.47 | 9 | 10 | 0.91 | 0.38 | 2.22 | 6 | 16 | 1.22 | 0.41 | 3.60 |
| Heptachlor | Never | 193 | 85 | 138 | 78 | Ref |  |  | 71 | 79 | Ref |  |  | 29 | 73 | Ref |  |  |
|  | M1 | 13 | 6 | 22 | 12 | 1.91 | 0.90 | 4.04 | 9 | 10 | 1.51 | 0.60 | 3.82 | 5 | 13 | 1.98 | 0.62 | 6.31 |
|  | M2 | 20 | 9 | 18 | 10 | 0.98 | 0.49 | 2.00 | 10 | 11 | 1.03 | 0.44 | 2.40 | 6 | 15 | 1.54 | 0.54 | 4.44 |
| DDT | Never | 182 | 81 | 141 | 79 | Ref |  |  | 75 | 82 | Ref |  |  | 31 | 78 | Ref |  |  |
|  | M1 | 17 | 8 | 24 | 13 | 1.35 | 0.67 | 2.75 | 10 | 11 | 1.02 | 0.42 | 2.46 | 6 | 15 | 1.22 | 0.41 | 3.58 |
|  | M2 | 27 | 12 | 14 | 8 | 0.49 | 0.23 | 1.06 | 6 | 7 | **0.35** | **0.13** | **0.99** | 3 | 8 | NA |  |  |
| Lindane | Never | 183 | 81 | 139 | 79 | Ref |  |  | 74 | 82 | Ref |  |  | 30 | 77 | Ref |  |  |
|  | M1 | 21 | 9 | 19 | 11 | 1.10 | 0.56 | 2.17 | 8 | 9 | 0.87 | 0.36 | 2.08 | 3 | 8 | NA |  |  |
|  | M2 | 23 | 10 | 17 | 10 | 0.89 | 0.45 | 1.78 | 8 | 9 | 0.76 | 0.32 | 1.84 | 6 | 15 | 1.39 | 0.50 | 3.88 |
| Toxaphene | Never | 204 | 91 | 160 | 90 | Ref |  |  | 81 | 90 | Ref |  |  | 36 | 90 | Ref |  |  |
|  | M1 | 10 | 4 | 9 | 5 | 1.09 | 0.42 | 2.81 | 6 | 7 | 1.43 | 0.49 | 4.19 | 3 | 8 | NA |  |  |
|  | M2 | 11 | 5 | 8 | 5 | 0.87 | 0.33 | 2.30 | 3 | 3 | 0.62 | 0.16 | 2.36 | 1 | 3 | NA |  |  |

^1^M1 = below the median, M2 = at or above the median intensity-weighted lifetime days.

^2^Odds Ratios (ORs) and 95% Confidence Intervals (CI) were calculated by multivariable logistic regression models adjusted for age at interview, state, overweight/obese, ever smoked, spring or summer season, current occupational pesticides use. NA=not applicable due to fewer than 5 exposed cases. Bolded values are statistically significant at p<0.05 level.
